# The value of hyperbaric oxygen therapy in postoperative care of subarachnoid hemorrhage

**DOI:** 10.1186/2045-9912-2-29

**Published:** 2012-12-15

**Authors:** Christoph J Griessenauer, Mohammadali M Shoja, Marios Loukas, R Shane Tubbs

**Affiliations:** 1 Division of Neurosurgery, Department of Surgery, University of Alabama at Birmingham, Birmingham, AL, USA; 2 Pediatric Neurosurgery, Children’s Hospital, Birmingham, AL, USA; 3 Department of Anatomical Sciences, School of Medicine, St. George's University, St. George, West Indies, Grenada

**Keywords:** Oxygen, Cerebral vasospasm, Subarachnoid hemorrhage

## Abstract

In this editorial, the issues related to the hyperbaric oxygen therapy and its utility in managing cerebral vasospasm in patients with subarachnoid hemorrhage is discussed.

## 

Cerebral vasospasm, defined as stenosis of intracranial arteries, is one of the main causes of morbidity and mortality in patients with subarachnoid hemorrhage (SAH). The incidence peaks around the seventh day after SAH and at that time some degree of vasospasm is evident on up to 70% of cerebral angiograms [1]. Symptoms related to vasospasm appear with at least a 50% reduction in arterial caliber [2] and occurs in about a quarter of patients with SAH [3]. It is an important cause of cerebral ischemia and leading, though potentially treatable, cause of morbidity and mortality after SAH [1]. Development of vasospasm is best predicted by the amount of subarachnoid blood early after SAH (the amount of bleeding is assessed by computed tomography (CT) scanning and is graded according to the Fisher CT grade) [4]. Gold standard for diagnosis is cerebral angiography [5]. Noninvasive imaging techniques including CT angiography, CT perfusion, or transcranial Doppler ultrasound (TCD) are frequently obtained, but generally less accurate. Currently, the mainstay of prevention and treatment includes optimization of cerebral blood flow through systemic administration of nimodipine, hyperdynamic therapy, and pharmacologic of mechanical angioplasty [2]. Notably, hyperbaric oxygen therapy stands as a potentially new, cost-effective and safe method for managing cerebral vasospasm [6,7]. Hyperbaric oxygen therapy not only protects the hypoperfused brain tissue from ischemia but also improves cerebral perfusion by reducing vasospasm.

Vasospasm creates a state of focal ischemia in the area irrigated by the stenotic vessel. Numerous studies have demonstrated a neuroprotective effect of hyperbaric oxygen in experimental animal models of focal ischemic brain injury and SAH [8]. Clinical trials of hyperbaric oxygen as a treatment for patients with symptomatic cerebrovascular disease and lacunar infarctions [9] and in prevention of neuropsychometric dysfunction after coronary bypass grafting [10] have shown promising results. Hyperbaric oxygen therapy is defined as a fraction of inspired oxygen administered at supra-atmospheric pressure in a hyperbaric oxygen chamber. Hyperbaric oxygen increases oxygen tension in the brain to levels where normal neuronal metabolism is maintained even in absence of hemoglobin delivered oxygen. Hyperbaric oxygen has variable effect on the cerebral blood flow and appears to reduce intracranial pressure [8]. Decreased brain infarction and improved neurobehavioral performance with hyperbaric oxygen therapy have been attributed to reduced blood–brain barrier breakdown, a decrease in inflammatory response, reduced oxidative and excitotoxic stress, decreased apoptosis and improved neural regeneration [1].

Transient exposure to hyperbaric oxygen appears to be benign in healthy human subjects even though extreme hyperbaric conditions above 4 to 5 atmosphere absolute (ATA) may result in brain oxygen toxicity and susceptibility to seizures [11]. Limitations of the hyperbaric oxygen therapy include its practicality and applicability in clinical practice given the necessity to have a hyperbaric oxygen chamber. Initiation of hyperbaric therapy in close temporal relationship to the onset of ischemia creates another challenge as delayed administration might actually be harmful [8] as in rodent models of transient brain ischemia, a delayed hyperbaric therapy aggravated the ischemic brain injury both histologically and clinically [12,13].

In a study [14], patients with aneurysmal SAH from an anterior circulation aneurysm were randomly assigned to receive at least 20 daily hyperbaric therapy (2 ATA) sessions lasting 60 minutes including pressurization and depressurization starting within one to three days from their aneurysm surgery. A total of 120 patients were included and randomized in equal proportions to hyperbaric therapy or control group. Primary endpoint was Glasgow Outcome Score at 6 months, and secondary endpoints included middle cerebral artery flow velocities measured by TCD, abnormal density in the operative region as a surrogate for cerebral infarction or edema, cases of symptomatic vasospasm, and Karnofsky Performance Scale (KPS). Symptomatic vasospasm was defined as development of a focal neurological deficit or deterioration in level of consciousness between the 3^rd^ and 14^th^ day after surgery associated with an increase in mean TCD velocity of 120 cm/sec in the investigated territory. The hyperbaric oxygen group performed favorably in terms of mean TCD velocities at days 7 and 14, smaller abnormal density volumes at days 7, 14, and 21, a smaller number of symptomatic vasospasm patients on days 7 and 14, higher KPS scores on day 21, and more patients with GOS 4 and 5 at 6 months. All these findings were statistically significant.

The utility of hyperbaric oxygen therapy in treatment of cerebral vasospasm sounds quite interesting and signals a new line of related studies to further underpin its advantages and disadvantages over current therapies. Current evidence of hyperbaric oxygen as a treatment of cerebral vasospasm is interesting. Ceverbral vasospasm is a potentially devastating complication of SAH and is urgently in need of a definitive treatment. Therefore, future randomized clinical trials are desired to investigate the utility of hyperbaric oxygen therapy in managing this condition.

## Competing interests

The authors declare that they have no conflict of interest in connection with this manuscript.
